# Body Fat Mediates Association between Active Living and Health among Adolescents

**DOI:** 10.3390/ijerph17165715

**Published:** 2020-08-07

**Authors:** Stanislava Stranavska, Daniela Husarova, Jiri Michal, Karol Gorner, Jaroslava Kopcakova

**Affiliations:** 1 Department of Physical Education and Sport, Faculty of Arts, Matej Bel University, 974 01 Banska Bystrica, Slovakia; Jiri.Michal@umb.sk (J.M.); Karol.Gorner@umb.sk (K.G.); 2 Department of Health Psychology and Research Methodology, Faculty of Medicine, P.J. Safarik University, 040 11 Kosice, Slovakia; daniela.husarova@upjs.sk (D.H.); jaroslava.kopcakova@upjs.sk (J.K.)

**Keywords:** physical activity, organized leisure-time activities, self-rated health, body fat percentage, adolescents, health behavior, Health Behaviour in School-aged Children (HBSC) study

## Abstract

The aim of this study was to explore the association of moderate-to-vigorous physical activity and organized leisure-time activities with self-rated health among adolescents and whether these associations are mediated by body fat percentage. We used data on 888 adolescents (mean age 12.97, SD 1.20, 56.0% boys) from the Health Behaviour in School-aged Children (HBSC) study conducted in 2018 in Slovakia. We used logistic regression models to examine associations within self-reported data (moderate-to-vigorous physical activity and organized leisure-time activities with self-rated health) and their mediation by anthropometric data (body fat percentage). The adolescents who were sufficiently physically active and with normal body fat were more likely to report good or excellent health (odds ratios—OR/95% confidence intervals—95% CI: 3.52/1.50–8.27 and 3.66/2.37–5.68). Similarly, the adolescents who were engaged in individual/team sport and with normal body fat were more likely to report good or excellent health (OR/95% CI: 2.04/1.31–3.17 and 3.66/2.37–5.68). Adjustment for body fat percentage reduced the association between moderate-to-vigorous physical activity and self-rated health by 27.6% and the association between leisure-time activities and self-rated health by 30.7%. Active living and normal body fat might contribute to better health in adolescence. Programs and efforts to increase physical activity and leisure-time activities in childhood and adolescence need to identify which aspects of these activities are important, effective, and crucial for the population of adolescents.

## 1. Introduction

Regular moderate-to-vigorous physical activity, as well as engagement in leisure-time activities, offers many benefits for physical and psychological health among adolescents [1,2]. Moderate-to-vigorous physical activity and organized leisure-time activities are positively linked to adolescents’ mental health and well-being, regardless of sociocultural and socio-economic differences, and any participation is generally better for their well-being than non-participation [1,3,4]. Moreover, sufficient physical activity might lead to better academic and cognitive performance among youth [5]. On the other hand, lower levels of physical activity might be associated with overweight and obesity, as well as poor health outcomes [5,6]. In addition, previous studies found that adolescents involved in organized leisure-time activities reported higher life satisfaction [7] and showed better psychological adjustment [8], school performance [9], and enhanced health and well-being [3,10]. Conversely, participants in organized leisure-time activities are less likely to be engaged in health-risk behaviors [11,12]. Therefore, the establishment of healthy patterns of organized leisure-time activities and regular physical activity during childhood is important for their health in adulthood. Kelleher et al. [13] found that adolescents with poor self-rated health reported more health complaints, lower levels of physical activity, and lower life satisfaction. In addition, previous studies showed a significant association between negative self-rated health and lower cardiorespiratory fitness, higher Body Mass Index, and higher body fat [14,15]. Therefore, a clearer understanding of the relationships between specific physical activity (e.g., sport participation), overall physical activity, and self-rated health might contribute to the development of specific preventive strategies focused on increasing the engagement in physical activity of adolescents [2].

Measuring total body fat percentage instead of Body Mass Index is considered to be more suitable as a measurement of weight because the measurement of total body fat percentage yields estimates of lean mass or fat mass [16]. Body fat percentage measurement is a suitable, but not routinely used, indicator of childhood obesity by the identification of excess body fat [17]. Childhood overweight and obesity has been among the most serious and alarming public health challenges within the last period [18], and their prevalence has tripled over the last three decades among adolescents [19].

The adolescents involved in organized sport activities spent significantly more daily time engaged in moderate-to-vigorous physical activity, mainly the girls [20], and it has influenced their health, fitness, and provided behavior benefits [21]. In addition, high-intensity physical activity may not lead to overall weight loss, but it may be associated with an increase in lean mass, instead of fat mass, as well as changes in body fat distribution [22]. Previous studies on adolescents have found that higher levels of moderate-to-vigorous physical activity or organized sport activities are associated with lower body fat percentages [23,24,25,26]. Based on these studies, we decided to take a closer look at how sufficient physical activity might be associated with health indirectly through objectively measured body fat among adolescents. Therefore, the aim of this study was to explore the association of moderate-to-vigorous physical activity and organized leisure-time activities with self-rated health among adolescents and whether these associations are mediated by body fat percentages among adolescents.

## 2. Materials and Methods

### 2.1. Sample and Procedure

We used self-reported data from the Health Behaviour in School-aged Children (HBSC) study conducted in 2018 in Slovakia. The HBSC study used a two-step sampling method to obtain a representative sample. In the first step, 140 larger and smaller elementary schools located in rural and urban areas from all regions of Slovakia were asked to participate. These were randomly selected from a list of all eligible schools in Slovakia obtained from the Slovak Institute of Information and Prognosis for Education. The school response rate (RR) was 77.85%. In the second step, we obtained data from 8405 adolescents from the fifth to ninth grades of elementary schools in Slovakia in the target group of 11–15 years old (mean age 13.43; 50.9% boys).

From the total HBSC study sample, 10% of the elementary schools were randomly selected for the purpose of anthropometric measurements. Finally, we obtained data from 888 adolescents from the fifth to ninth grades of 12 elementary schools in Slovakia in the target group of 11–15 years old (mean age 12.97; SD 1.20; 56.0% boys).

The parents were informed about the study via the school administration, and passive consent was obtained from them. If they disagreed with the participation of their children in the study, they could opt out. Participation in the study was fully voluntary and anonymous.

The study was approved by the Ethics Committee of the Medical Faculty at the P.J. Safarik University in Kosice (16N/2017).

### 2.2. Measures

#### 2.2.1. Anthropometric Data

Body fat percentage (%) was measured by Bioimpedance Body Composition Analysis (BIA) with an InBody 230 (Biospace Co., Ltd., Seoul, Korea). The analysis was carried out according to the manufacturer’s instructions [27]. The adolescents were instructed to be dressed in a t-shirt and trousers or skirt prior to measurement. The starting weight was set to −0.5 kg, considering the fact that we did not weigh the school-aged children in their underwear. The boys and girls with a proportion of body fat over 25% and 30%, respectively, were considered to be adolescents with excess body fat vs. adolescents with normal body fat (under these cut-offs) [16,28].

#### 2.2.2. Self-Reported Data

Active living among adolescents was assessed by two measures: moderate-to-vigorous physical activity and engagement in sport organized leisure-time activities.

Moderate-to-vigorous physical activity was measured by an item asking adolescents about the number of days over the past week that they were physically active for at least 60 min per day. The question was preceded by an explanatory text that defined moderate-to-vigorous activity as “any activity that increases your heart rate and makes you get out of breath some of the time”, offering examples of such activities (running, inline skating, cycling, dancing, swimming, ice skating, etc.) [2,29]. The responses concerned 0–7 days per week and were classified as sufficient (physical activity for 7 days) vs. insufficient (physical activity for 0-6 days), based on the WHO recommendation [30]. A test-retest study demonstrated moderate agreement and a similarly moderate correlation after dichotomization of this instrument [31].

Engagement in organized leisure-time activities was assessed by the question: “In your leisure time, do you do any of these organized activities? Organized activities refer to those activities that are done in a sport or another club or organization.” The following categories of organized activities were then listed: organized team sports, organized individual sports, art school/club (e.g., playing a musical instrument, singing, dance, drama), youth organizations (e.g., Scouts, Sokol), leisure centers or after-school clubs (e.g., chess, model building, debate clubs), church meeting or singing (e.g., Salesians) [2,29]. For the purpose of our study, we focused on adolescents who indicated engagement in team or individual sports. Then, we computed the total score from the positive answers to create a composite variable. Furthermore, we dichotomized the composite variable to create a binary variable with two categories: (1) no activity; (2) individual or team sport or both. A test-retest study demonstrated the substantial reliability of the instrument [32].

Self-rated health was measured by the question “Would you say your health is….?” [2] with the following response options: excellent, good, fair, or poor. The responses were dichotomized into two categories: (1) excellent or good health; (2) fair or poor health [29].

### 2.3. Statistical Analyses

First, we described the sample using descriptive statistics. Second, we assessed the association between moderate-to-vigorous physical activity, as well as organized leisure-time activities (used as categorical variable) separately, and body fat percentage (dichotomized) using logistic regression models that were adjusted for age (continuous) and gender. Third, we assessed the association between moderate-to-vigorous physical activity, as well as organized leisure-time activities and body fat percentage separately, and self-rated health using logistic regression models, adjusted for age and gender. We reported the odds ratio (OR) with a 95% confidence interval (CI). Model 1 tested the association of moderate-to-vigorous physical activity, organized leisure-time activities, and body fat percentage each separately with self-rated health. Model 2 was adjusted for body fat percentage. The degree of reduction in the ORs’ was computed using the formula: (OR (crude) −OR (adjusted))/(OR (crude) − 1) × 100%. All of the statistical analyses were performed using SPSS v.23.0 for Windows, except for the calculation of the statistical significance of the mediation effect, which was performed in R 4.0.2 for Windows using the mediation package [33].

## 3. Results

The background characteristics of the sample are presented in Table 1. Only 20% of the adolescents reported sufficient moderate-to-vigorous physical activity, and around 37% of them were not engaged in any sport activity. Moreover, 23% of adolescents were indicated as adolescents with excess body fat.

Next, the association between moderate-to-vigorous physical activity, as well as organized leisure-time activities separately, and body fat percentage was found to be statistically significant. The adolescents who were sufficiently physically active and were engaged in individual/team sports were more likely to have normal body fat (Table 2).

Finally, associations of moderate-to-vigorous physical activity and body fat percentage with self-rated health, separately, were statistically significant. The adolescents who were sufficiently physically active and with normal body fat were more likely to report good or excellent health (Table 3 Model 1). The association between moderate-to-vigorous physical activity and self-rated health remained significant after the additional adjustment for body fat percentage (Table 3 Model 2). The change in the odds ratio (OR) confirmed the mediating role of body fat percentage in the association between moderate-to-vigorous physical activity and self-rated health, and the degree of reduction was 27.6% (Table 3). We tested the significance of the indirect effect using the bootstrapping procedure. Unstandardized indirect effects were computed for each of 5000 bootstrapped samples, and the 95% confidence interval was computed by determining the indirect effects at the 2.5th and 97.5th percentiles. The bootstrapped, unstandardized indirect effect was 0.026, and the 95% confidence interval ranged from 0.009–0.03. Thus, the indirect effect was statistically significant (*p* < 0.001).

Similarly, the associations of organized leisure-time activities and body fat percentage with self-rated health, separately, were statistically significant. The adolescents who were engaged in individual/team sports and with normal body fat were more likely to report good or excellent health (Table 4 Model 1). The association between organized leisure-time activities and self-rated health remained significant after the additional adjustment for body fat percentage (Table 4 Model 2). The change in the odds ratio (OR) confirmed the mediating role of body fat percentage in the association between organized leisure-time activities and self-rated health, and the degree of reduction was 30.7% (Table 4). The bootstrapped, unstandardized indirect effect was 0.023, and the 95% confidence interval ranged from 0.012—0.04. Thus, the indirect effect was statistically significant (*p* < 0.001).

## 4. Discussion

We explored the association of moderate-to-vigorous physical activity and organized leisure-time activities with self-rated health among adolescents and whether these associations are mediated by body fat percentage among adolescents. We found that adolescents who were sufficiently physically active and with normal body fat were more likely to report good or excellent health. Similarly, our results indicate that adolescents who were engaged in individual/team sport and with normal body fat were more likely to report good or excellent health.

Our findings show that moderate-to-vigorous physical activity, as well as organized leisure-time activities, was associated with normal body fat. Evidence has shown that the prevalence of adolescents with normal weight, as well as with overweight and obesity, differs across different countries and regions, but it is generally known that overweight and obesity is higher in boys than in girls, adolescents with lower socio-economic status than adolescents with higher socio-economic status, and among younger than among older adolescents [18]. At the same time, despite the importance of physical activity at all ages, including youth, physical activity among adolescents over the past few decades has significantly declined [34]. In particular, more than two-thirds of adolescents aged 11–17 years do not meet the recommendation of 60 min of moderate-to-vigorous physical activity every day [30,35]. Similarly, the reason for the declining of participation in organized leisure-time activities with age and why adolescents drop out of organized leisure-time activities is still questionable, but is present within adolescents nevertheless [2,3].

We found that adolescents who were sufficiently physically active and with normal body fat were more likely to report good or excellent health. Our results are in line with the findings of the 2018 Physical Activity Guidelines Advisory Committee Scientific Report [35], demonstrating a positive association between physical activity, self-rated health, and quality of life. Previous findings have revealed that sufficient physical activity is associated with good or excellent self-rated health among adolescents [36,37,38] and more time spent in every type of physical activity, including light-intensity physical activity (e.g., leisurely walking/sports, moderate-to-vigorous physical activity, vigorous physical activity etc.), has been associated with a lower risk of cardiovascular disease, increased socialization, and, thus, related to self-rated health [35,39].

Our results indicate that adolescents who were engaged in individual/team sport and with normal body fat were more likely to report good or excellent health. Previous studies also showed that engagement in organized leisure-time activities is associated with better life satisfaction, excellent self-rated health, and lower amounts of psychological complaints [40,41]. In addition, participation in organized leisure-time activities, such as sports, arts, and youth organizations, has been associated with both better psychosocial adjustment, including better school performance and attainment [42,43], and enhanced health and well-being [3,7]. In addition, organized sport activities contribute to the proportion of youth meeting physical activity recommendations and, based on the results of Marques et al. [26], participation in organized sport did not necessarily contribute to the classification of adolescents with overweight or obesity who were also identified by excess body fat.

There are several strengths and limitations that need to be considered in this study. The main strength of our study was using objectively measured body fat percentage as an appropriate tool for identifying excess body fat and overweight or obesity among adolescents [44]. Another strength of this study is its representativeness of the sample and the comparability of our data with the international data within the HBSC study. On the other hand, the main limitation was using self-reported data on physical activity and self-rated health, with the reliability of self-reported physical activity measures being discussed in the literature. However, they have been reported to have acceptable test-retest reliability, and rank time spent on this activity accurately [31,45]. In addition, self-rated health of adolescents may be affected also by other factors than moderate-to-vigorous physical activity, organized leisure-time activities, body fat percentage, age, and gender. Another limitation was the cross-sectional design of this study, which did not allow us to formulate a conclusive statement regarding the causality of our results. Therefore, our findings need to be confirmed in longitudinal studies.

## 5. Conclusions

Sufficient physical activity and engagement in organized leisure-time activities were found to be directly associated with self-rated health. Moreover, indirect associations through body fat percentage were also confirmed. Physical and leisure activities might be used as a treatment for several chronic diseases associated with self-rated health [46]. Our results imply that active living—including regular physical activity and engagement in organized leisure-time activities—and normal body fat might contribute to better health in adolescence. Therefore, programs and efforts to increase physical activity and leisure-time activities in childhood and adolescence need to identify which aspects of these activities are important, effective, and crucial for the population of adolescents.

## Figures and Tables

**Table 1:** Description of the sample.

Characteristics	*n* (%)
Gender	
Boys	497 (56.0)
Girls	391 (44.0)
Age	
11 years old	146 (16.4)
12 years old	199 (22.4)
13 years old	208 (23.4)
14 years old	206 (23.2)
15 years old	129 (14.5)
Organized leisure-time activities	
Individual or team sport	544 (63.3)
No activities	316 (36.7)
Moderate-to-vigorous physical activity	
Sufficient	179 (20.2)
Insufficient	705 (79.8)
Body Fat Percentage	
Normal	684 (77.0)
Excess	204 (23.0)
Self-rated health	
Good and excellent	773 (87.3)
Fair and poor	112 (12.7)

Note: Only valid percentages are presented; missing values: organized leisure-time activities = 28 (3.2%), moderate-to-vigorous physical activity = 4 (0.5%), self-rated health = 3 (0.3%).

**Table 2:** The association of organized leisure-time activities and moderate-to-vigorous physical activity with body fat percentage, adjusted for age and gender (odds ratios—OR/95% confidence interval—95%CI).

Studied Variables	Body Fat Percentage
	OR (95%CI)
Moderate-to-vigorous physical activity	
Insufficient	Ref.
Sufficient	2.93 (1.78–4.83) ***
Organized leisure-time activities	
No activities	Ref.
Individual or team sport	2.36 (1.69–3.30) ***

Note: ***—*p* < 0.001; Ref.- reference group.

**Table 3:** The association of moderate-to-vigorous physical activity with self-rated health, adjusted for age and gender, and additionally adjusted for body fat percentage from logistic regression models (odds ratios—OR/95% confidence interval—95%CI).

Studied Variables	Model 1	Model 2
	OR (95%CI)	OR (95%CI)
Moderate-to-vigorous physical activity		
Insufficient	Ref.	Ref.
Sufficient	4.48 (1.92–10.42) **	3.52 (1.50–8.27) **
Body fat percentage		
Excess	Ref.	Ref.
Normal	4.28 (2.81–6.52) ***	3.83 (2.50–5.87) ***
* Change of OR for weight		27.6%

Note: ***—*p* < 0.001, **—*p* < 0.01; Ref.- reference group; Model 1—The association of moderate-to-vigorous physical activity and body fat percentage with self-rated health separately; Model 2—The mediating effect of body fat percentage on association of moderate-to-vigorous physical activity with self-rated health; *—Decrease in OR for body fat percentage due to adjustment, compared with Model 1 (in Model 2).

**Table 4:** The association of organized leisure-time activities with self-rated health, adjusted for age and gender, and additionally adjusted for body fat percentage from logistic regression models (odds ratios—OR/95% confidence interval—95%CI).

Studied Variables	Model 1	Model 2
	OR (95%CI)	OR (95%CI)
Organized leisure-time activities		
No activities	Ref.	Ref.
Individual or team sport	2.50 (1.63–3.82) ***	2.04 (1.31–3.17) **
Body fat percentage		
Excess	Ref.	Ref.
Normal	4.28 (2.81–6.52) ***	3.66 (2.37–5.68) ***
* Change of OR for weight		30.7%

Note: ***—*p* < 0.001, **—*p* < 0.01; Ref.- reference group; Model 1—The association of organized leisure-time activities and body fat percentage with self-rated health separately; Model 2—The mediating effect of body fat percentage on the association of organized leisure-time activities with self-rated health; *—Decrease in OR for body fat percentage due to adjustment, compared with Model 1 (in Model 2).
